# Estimated maximal and current brain volume predict cognitive ability in old age

**DOI:** 10.1016/j.neurobiolaging.2013.05.015

**Published:** 2013-12

**Authors:** Natalie A. Royle, Tom Booth, Maria C. Valdés Hernández, Lars Penke, Catherine Murray, Alan J. Gow, Susana Muñoz Maniega, John Starr, Mark E. Bastin, Ian J. Deary, Joanna M. Wardlaw

**Affiliations:** a Brain Research Imaging Centre, Neuroimaging Sciences, University of Edinburgh, Edinburgh, UK; b Centre for Cognitive Ageing and Cognitive Epidemiology, University of Edinburgh, Edinburgh, UK; c Scottish Imaging Network, A Platform for Scientific Excellence (SINAPSE) Collaboration, Edinburgh, UK; d Centre for Clinical Brain Sciences, University of Edinburgh, Edinburgh, UK; e Department of Psychology, University of Edinburgh, Edinburgh, UK; f Alzheimer Scotland Dementia Research Centre, University of Edinburgh, Edinburgh, UK

**Keywords:** Aging, Structural brain imaging biomarkers, Brain volume, Life course cognitive ability, IQ

## Abstract

Brain tissue deterioration is a significant contributor to lower cognitive ability in later life; however, few studies have appropriate data to establish how much influence prior brain volume and prior cognitive performance have on this association. We investigated the associations between structural brain imaging biomarkers, including an estimate of maximal brain volume, and detailed measures of cognitive ability at age 73 years in a large (N = 620), generally healthy, community-dwelling population. Cognitive ability data were available from age 11 years. We found positive associations (*r*) between general cognitive ability and estimated brain volume in youth (male, 0.28; females, 0.12), and in measured brain volume in later life (males, 0.27; females, 0.26). Our findings show that cognitive ability in youth is a strong predictor of estimated prior and measured current brain volume in old age but that these effects were the same for both white and gray matter. As 1 of the largest studies of associations between brain volume and cognitive ability with normal aging, this work contributes to the wider understanding of how some early-life factors influence cognitive aging.

## 1 Introduction

As individuals age, some degree of decline is typically observed in the mean test scores of cognitive abilities such as reasoning, memory, processing speed, and spatial ability (Ghisletta et al., 2012; Salthouse, 2010). A review by Plassman et al. (2010) found that, although there was evidence for other factors, health problems, negative lifestyle choices (especially smoking), and possession of the *APOE*-ε4 allele were consistently associated with an increased risk of age-related cognitive decline.

Important factors not considered by Plassman et al. (2010) are decline or changes in brain tissue volume. This is surprising, as larger brain volume, estimated by magnetic resonance imaging (MRI), has been associated with higher intelligence, with reported associations ranging from 0.33 to 0.42 across studies (McDaniel, 2005; Miller and Penke, 2007; Rushton and Ankney, 2009). Furthermore, studies have shown that there are direct links between reduction in brain tissue volume and cognitive decline (Sluimer et al., 2008), that decline in brain tissue volume and increased cerebrospinal fluid (CSF) longitudinally are associated with lower cognitive performance (Cardenas et al., 2011), and that tissue loss is accelerated in individuals with mild cognitive impairment compared with the normal decline with aging (Driscoll et al., 2009).

However, several factors remain unclear in the associations between brain status and cognitive ability in later life. First, research has been equivocal as to whether prior maximal brain size, as estimated by intracranial volume (ICV), or current brain status, as measured by current tissue volume, is the stronger predictor of later life cognitive ability. Maximal brain volume is established in late childhood and thereafter is reflected broadly in the internal size of the cranial cavity (Wolf et al., 2003), which can be measured using ICV from structural MRI. The volume of the intracranial cavity is directly related to brain growth in youth, and although brain volume begins to decline in early adulthood, the cranial cavity is considered to remain relatively stable thereafter (Blatter et al., 1995) thus representing an “archeological” estimate of maximal brain size in youth that is available to studies at older ages. A study looking at various volume measurements, including ICV and total brain volume (TBV), across a large age range (19 months to 80 years of age) found that ICV increased with brain volume throughout childhood and early adolescence but that, between the ages of 16 and 80 years, the brain volume had significantly decreased (Courchesne et al., 2000) whereas the ICV showed no change. Therefore, although brain tissue volume decreases with age, ICV remains approximately unchanged after brain development ceases in youth, the brain achieves its maximum size, and skull sutures fuse, and as such, may be considered as an estimate for prior or maximal brain size.

A number of studies have shown positive associations between intracranial volume (ICV) and cognitive ability in later life. MacLullich et al. (2002) report positive significant correlations, ranging from 0.26 to 0.39, between ICV and several individual cognitive ability tests. Shenkin et al. (2009) found, in 107 general healthy older adults between 75 and 81 years of age, that whole brain volume accounted for little (<1%) variance in general cognitive ability, whereas intracranial area (a proxy measure of ICV), explained 6.2% of the variance. More recently, Farias et al. (2012) found that ICV and current brain volumes (i.e., total brain, hippocampal, and white matter lesions) in older adults were associated with different cognitive domains, but that ICV correlated with cognitive variables after the other brain volume measures were accounted for. These findings suggest that maximal brain size is an important factor in understanding late-life cognitive ability.

A possible explanation for the associations found between maximal brain size, measured using ICV, and late-life cognitive ability could be the passive cerebral reserve hypothesis (Stern, 2009). It has been suggested that possessing a larger brain provides some resistance against neurodegeneration in later life, consequently attenuating cognitive decline (Mori et al., 1997). The concept of passive cerebral reserve has been proposed to explain individual differences in brain aging, or more specifically, to explain the differential effects seen in individuals who display the same proportional amount of brain aging (Staff et al., 2004; Stern, 2009). It may be the case that positive associations between ICV and good cognitive performance in later life reflect an individual's brain's ability to withstand the normal aging process.

Despite research evidence showing prior cognitive ability to be the strongest predictor of later-life cognitive ability (Deary et al., 2012), few studies investigating the association between contemporaneous brain volumes and cognitive ability control for it. Of those studies that have (Murray et al., 2011; Staff et al., 2004), no support has been found for the prediction of contemporaneous cognitive performance by brain volumes when controlling for prior ability.

Brain tissue volume decline in the normal aging brain is not uniform; gray matter may begin declining in early adulthood and follow a fairly linear pattern thereafter, whereas white matter volume is said to increase until around middle age and then start to decrease (Ge et al., 2002; Ziegler et al., 2010). Findings that prefrontal white matter volume decline is significantly greater than gray matter volume decline in old age, particularly in the 9th decade, suggests that white matter is more vulnerable to normal aging (Salat et al., 1999). Further, research has suggested that the divergence between volumetric changes in gray and white matter tissue volume is reflected in the associations between these tissues and cognitive performance (Raz et al., 2010).

To provide an examination of the associations between brain size and cognitive ability in aging, in the present study we tested whether maximal brain size in youth (as estimated by ICV) and current brain tissue volume significantly contributed to cognitive ability in later life. We then tested the contributions to both current cognitive ability and lifetime change in cognitive ability of white and gray matter volumes separately. Overall, we aimed to provide a thorough assessment of the relative contributions of broad measures of the brain and cognitive performance in a large, age-homogenous sample of generally healthy older adults.

## 2 Method

### 2.1 Participants

The Lothian Birth Cohort 1936 (LBC1936) is a longitudinal study of cognitive aging, comprising individuals who mostly took part in the Scottish Mental Survey 1947 (SMS1947), and who were resident in Edinburgh and its surrounding area (the Lothians) at about age 70 years. The recruitment, brain imaging, and cognitive testing protocols for the LBC1936 have been reported previously in detail (Deary et al., 2007; Wardlaw et al., 2011).

The current study uses data from the second wave of testing, in which 866 participants (mean age = 72.5 years, SD = 0.7 years) returned for a second wave of cognitive testing. Of those returning, 700 completed an MRI scan, of which 672 completed all sequences necessary to measure brain volumes for this study. Of the participants, 41 were excluded for incomplete cognitive data (age 11 IQ, 36; individual cognitive assessments at Wave 2 assessment, 5), and 11 participants were excluded as they scored ≤24 on the Mini Mental State Examination (MMSE). A score of ≤24 on the MMSE is a widely used clinical cut-off considered to be indicative of possible pathological cognitive impairment (Folstein et al., 1975). The final sample consisted of 620 adults (327 male, 52.7%).

Based on recent studies (O'Bryant et al., 2008; Stephan et al., 2010), and to test the robustness of the models described below, we also estimated all models using a more conservative MMSE cut-off excluding all those with scores ≤28. This was done to ensure that the results were not overly influenced by cases with lower MMSE scores, and thus potentially also those with mild cognitive impairment or early stages of dementia. In this secondary analysis, a further 83 participants (male, 57; female, 26) were removed, resulting in a sample of 537 (270 males, 50.3%).

### 2.2 Cognitive testing

Cognitive ability at age 11 years was assessed using the Moray House Test No. 12 (MHT) which participants took as part of the SMS1947. The MHT includes a range of cognitive ability items (following directions, same-opposites, word classification, analogies, practical items, reasoning, proverbs, arithmetic, spatial items, mixed sentences, and cypher decoding) and is a validated measure of general intelligence (Deary et al., 2007). The age 11 MHT scores were converted into an IQ-type score (age 11 IQ; mean =100, SD = 15), after controlling for age in days at which the test was taken.

Later-life cognitive ability was measured using 6 non-verbal subtests of the Wechsler Adult Intelligence Scale (WAIS-III^UK^, Wechsler, 1998): 2 subtests of working memory (Letter Number Sequencing and Digit Span Backward), 2 subtests of speed of processing (Symbol Search and Digit Symbol), and 2 tests of nonverbal reasoning (Block Design and Matrix Reasoning).

### 2.3 Image acquisition

Structural MRI data were obtained from a GE Signa Horizon HDxt 1.5 T clinical scanner (General Electric, Milwaukee, WI, USA) using a self-shielding gradient set with maximum gradient strength of 33 m/Tm, and an 8-channel phased-array head coil. The examination included a T2W, T2_W and FLAIR axial scans, a high-resolution T1W volume sequence acquired in the coronal plane, axial T1W fast-spoiled gradient echo (FSPGR) sequences with 21 and 121 flip angles for quantitative T1-mapping, and 2 standard spin echo sequences acquired with and without an MT pulse applied 1 kHz from the water resonance frequency for MT-MRI (Wardlaw et al., 2011).

### 2.4 Image analysis

All image analysis was performed by trained analysts blinded to participant information.

The ICV included the contents within the inner skull table with its inferior limit in the axial slice just superior to the tip of the odontoid peg at the foramen magnum and superior to the inferior limits of the cerebellar tonsils (Wardlaw et al., 2011). The ICV, which includes brain tissue, cerebrospinal fluid (CSF), veins and dura, was obtained semi-automatically using the T2*W sequence, with the Object Extraction Tool in Analyze 9.0 (Mayo Clinic, Analyze 9.0. Analyze Direct, Inc Mayo Clinic) providing an initial segmentation that was manually edited to remove erroneous structures.

CSF, white matter lesions, and white and gray matter were extracted using MCMxxxVI (Valdés Hernández et al., 2010): a semi-automatic multispectral segmentation technique that uses color fusion to enhance tissue differentiation, and minimum variance quantization to dither the color spectrum and to create binary masks of the tissue or lesion in question. The combination of T2*W and FLAIR mapped in red and green, respectively, were used to extract CSF (identified in red color) and white matter lesions (identified in yellow). The CSF masks were then subtracted from the ICV to provide a measure of total brain tissue volume (TBV). The same method was used to produce white matter masks, but fusing T2W and T1W volumes (in red and green, respectively). In this color and sequence combination, healthy white matter was identified in bright green, differing from gray matter and white matter lesions. Gray matter masks were calculated by subtracting the white matter masks and white matter lesion binary masks from the brain tissue masks created previously.

### 2.5 Statistical analysis

All models were estimated using multi-group structural equation modeling (MG-SEM). This type of modeling has the advantage of modeling latent constructs, and the associations between them, taking account of measurement error, while providing formal tests of their equivalence across groups. As a result, multi-group structural equation modeling provides robust estimates of associations. All models were estimated in Mplus 6.0 (Muthen and Muthen, 2010) using maximum likelihood estimation.

For the current study, we used the multi-group model to estimate parameters for males and females separately. Sex is known to be one of the largest sources of variability in overall head and brain size, and, given our relatively large sample, we chose to model sex differences directly rather than simply include sex as a covariate. Input data for all models were standardized residuals after regressing out, and thus controlling for, variance associated with age in days. The exception to this was age 11 IQ, which is an age-standardized IQ type score. Despite the narrow age range of the current cohort, age in days still accounted for significant (*p <* 0.05) amounts of variance in brain tissue volume, white matter volume, gray matter volume, block design, digit symbol coding, symbol search, and matrix reasoning; demonstrating the importance of accounting for the effects of age.

#### 2.5.1 Model specification

First, it is important to state that, in all analyses, TBV, white matter (WM) and gray matter (GM) were standardized residuals controlling for ICV. In model 1, we included ICV and TBV as predictors of general cognitive ability (g) to assess the degree of association between measures of maximal brain size, current brain status and current cognitive ability. Next, we included age 11 IQ as a predictor of g to assess whether ICV and TBV remain significant predictors of current ability, controlling for past ability. As such, we were asking whether ICV and TBV also predict change in cognitive ability over the life course. In model 2, we follow the same sequence of analyses, but replace TBV with WM and GM volumes, to test whether associations with specific tissue types are consistent with g in both males and females.

#### 2.5.2 Measurement invariance

Before testing the equivalence of the regression parameters across males and females, measurement invariance was established for the latent constructs. Measurement invariance ensures that the latent constructs are equivalent across groups, and is required to make meaningful interpretations of model parameters across groups (French and Finch, 2006). We established configural invariance (equivalence of the pattern of factor loadings), and metric invariance (degree of factor loadings), for both g and processing speed.

Once measurement invariance has been established, parameters of interest within the models can be fixed to equivalence, and the plausibility of this constraint is tested using the difference in χ^2^ for the appropriate number of degrees of freedom.

#### 2.5.3 Model evaluation

In SEM, the degree to which a model conforms to the data is assessed using model fit indices. We adopted cut-off points based on a review (Schermelleh-Engel et al., 2003) of ≤0.05 for the standardized root mean square residual (SRMR), ≤0.06 for the root mean square error of approximation (RMSEA), and ≥0.95 for the Tucker-Lewis Index (TLI) and Comparative Fit Index (CFI). In establishing whether the assumptions of measurement invariance for the latent constructs hold, we follow Chen (2007) and suggest changes in CFI of −0.01 or less combined with changes in RMSEA ≤0.015 support measurement invariance.

## 3 Results

Descriptive statistics are shown in Table 1. All variables were approximately normally distributed with no values for skew exceeding ±0.90, or values for kurtosis exceeding ±1.17 in either the male or female participants.

Table 2 contains the uncorrected bivariate correlations. The significant positive correlations between the cognitive ability tests in males (r = 0.29–0.65) and females (r = 0.20–0.58), supports the modeling of latent cognitive ability factor, g. There were universally positive correlations between cognitive ability tests and total brain tissue volume (male r = 0.18–0.34; female r = 0.10–0.25), and white (male r = 0.13–0.26; female r = 0.04–0.18) and gray matter (male r = 0.04–0.26; female r = 0.01–0.13) volumes. Age showed several significant associations with both cognitive and brain volume variables in males (r = −0.18 to 0.13) and females (r = −0.23 to 0.11), supporting its inclusion as a covariate, despite the narrow age range in this cohort.

Table 2 also provides the associations of cognitive ability from youth and old age with ICV and brain size. To the extent that ICV may be considered an estimate of maximal brain size in youth, the correlation between ICV and age 11 IQ provides an estimate of brain–cognition associations in youth. In the current sample, these estimates are 0.28 (*p* < 0.001) for males and 0.12 (*p <* 0.05) for females. The correlations between latent g and concurrent brain volume provide similar contemporaneous associations at age 73 years. In the current sample, the g-brain volume correlation is 0.27 (*p* < 0.001) for males and 0.26 (*p* < 0.001) for females, highly comparable to the ICV–age 11 IQ association.

Total brain volume and white matter volume have several moderately significant associations with individual cognitive ability subtests in both males and females, whereas the associations with gray matter volume are largely small and nonsignificant. However, the correlations presented in Table 2 are raw correlations, uncorrected for ICV and age and as such, differ to some extent from the estimates presented in the final models.

Finally, Table 2 also provides estimates of the associations between ICV and concurrent brain volume, which correlate highly in both males and females (0.82, *p* < 0.001 and 0.83, *p* < 0.001, respectively). Gray matter shows weaker associations with both ICV than did white matter in males (ICV, 0.36 vs. 0.51) and females (ICV, 0.42 vs. 0.51).

Before testing the main models, we first established measurement invariance in the measurement model for g across males and females. The model showed excellent fit to the data (χ^2^ = 22.73(12), *p* < 0.05; CFI = 0.99; TLI = 0.97; RMSEA = 0.054; SRMR = 0.023), and the difference in model fit across the configural and metric invariance models fell within the suggested range of model fit (Δχ^2^ = 7.21(5), *p* > 0.05; ΔCFI = 0.00; ΔRMSEA = −0.004). The measurement model was therefore considered to be invariant across sex at the metric level, with all subsequent models run with the invariance constraints in place.

**Fig. 1:**
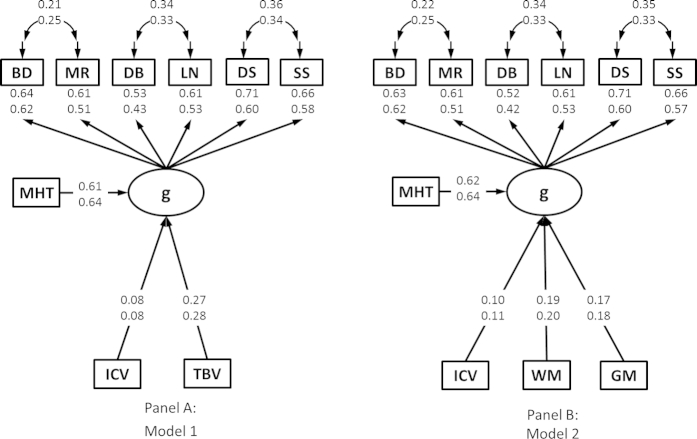
Structural diagram for models 1 and 2. All variables are residuals controlling for age. All values are standardized parameter estimates. Estimates are taken from the final models with parameters constrained across groups. Parameter estimates are presented for males (top) and females (bottom) separately. Note: No differences between male and female models were significant. All values are significant at a minimum of *p* < 0.05. Model fit for model 1 (χ^2^ = 59.59(53), *p* = 0.25; CFI = 1.00; TLI = 0.99; RMSEA = 0.020; SRMR = 0.034) and model 2 (χ^2^ = 88.42(64), *p* < 0.05; CFI = 0.98; TLI = 0.98; RMSEA = 0.035; SRMR = 0.036) was excellent. Abbreviations: BD, block design; DB, digit span backward; DS, digit symbol coding; GM, gray matter volume; ICV, intracranial volume; LN, letter–number sequencing; MHT, age 11 MHT IQ score; MR, matrix reasoning; SS, symbol search; TBV, total brain volume; WM, white matter volume.

Models 1 and 2 displayed excellent fit to the data (see Fig. 1 for final model fits). When all paths in the final models were sequentially constrained to equivalence across males and females, no χ^2^ differences reached statistical significance (Δχ^2^ ≥ 3.84, *p <* 0.05). Therefore, in the current sample, there were no significant differences in the magnitude of parameter estimates in the male and female models.

For model 1, we first tested a model including only ICV and TBV, but not prior cognitive ability. Both ICV and TBV were significant predictors of current cognitive ability in both males (ICV = 0.19, *p <* 0.001; TBV = 0.30, *p <* 0.001) and females (ICV = 0.21, *p <* 0.001; TBV = 0.30, *p <* 0.001). Combined ICV and TBV accounted for approximately 14% of the variance in current cognitive ability.

Next, we included prior ability (MHT) as a predictor of current ability level (Fig. 1A). Prior ability was the strongest predictor of current ability (males, 0.61, *p <* 0.001; females, 0.64, *p <* 0.001). ICV and TBV remained significant predictors of current cognitive ability; however, the magnitude of the association with ICV dropped to 0.08 (*p <* 0.05). In total, MHT, ICV, and TBV accounted for approximately 52% of the variance in later life cognitive ability.

Similarly, for model 2, we first tested a model excluding prior ability. In this model, ICV (males, 0.22, *p <* 0.001; females, 0.23, *p <* 0.001), WM (males, 0.22, *p <* 0.001; females, 0.21, *p <* 0.001), and GM (males, 0.18, *p <* 0.001; females, 0.18, *p <* 0.001) were all significant predictors of current cognitive ability accounting for a combined 11% of the variance in cognitive ability. Including prior ability (Fig. 1B) the magnitude of the association with ICV dropped (male, 0.10, *p <* 0.05; female, 0.11, *p <* 0.05), but remained significant. The magnitudes of the associations with WM and GM were approximately equal. Overall, in model 2, MHT, ICV, GM, and WM accounted for approximately 48% of the variance in current cognitive ability.

There were no substantive differences in either model 1 or 2 when models were re-estimated using the subsample of participants scoring ≥28 on the MMSE (n = 537). The regression paths of ICV to g in model 1 failed to reach significance in the reduced sample, but the parameter estimates were identical at the second decimal place, indicating the lack of significance is due to a reduction in power with sample size.

### 3.1 Life course stability of ICV

The models discussed above assume that the stability of ICV maximal brain size is reached in youth. To provide some support for this assumption, we present data from a freely available MRI data set (http://www.oasis-brains.org) of 416 healthy adults with ages ranging from 18 to 96 years. First, ICV and TBV are significantly correlated in youth (18–28 years; r = 0.85, n = 135, *p* = 0.00) showing that ICV is a good marker of individual differences in TBV in younger adults. As displayed in Table 3, when grouped by decade, mean ICV differs between the youngest (18–28 years old) and oldest group (84–96 years old) by 65 cm^3^, which is less than the standard deviation of 158 cm^3^ measured across the whole sample (18–96 years old). The percentage mean TBV, expressed as a percentage of ICV, shows a decrease of 14.1% between the youngest (84.7%) and oldest groups (70.6%), where the standard deviation across the whole group is 6%.

**Fig. 2:**
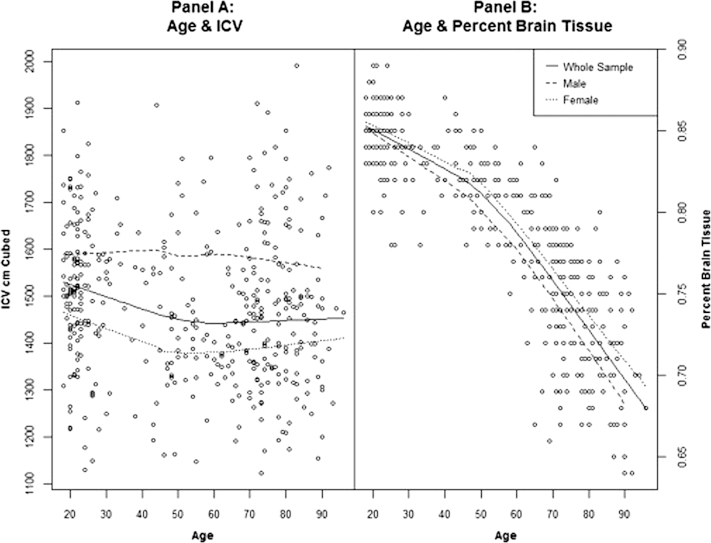
Plot depicting the stability of ICV from youth to old age (A) and the decline in the percentage of brain tissue in ICV from youth to old age (B). Data (N = 416) were taken from an open source imaging data base (http://www.oasis-brains.org). Data are plotted for the whole sample and for males and females separately.

Fig. 2 plots ICV (A) and TBV (B) by age for the whole sample, males and females. In all cases, ICV remains broadly stable across age, whereas TBV declines with age in an approximately equivalent manner in the whole sample, males and females.

## 4 Discussion

The current findings suggest that cognitive ability at age 73 years is dependent, in part, on prior cognitive ability, prior or maximal brain size, and current brain tissue volume. The study also finds that there were generally similar, modest-sized cross-sectional associations between brain size and cognitive ability in childhood and old age, although an estimate (ICV) had to be used for brain size in youth.

Across models, current brain tissue volume was a stronger predictor of later-life cognitive ability (both with and without controlling for past ability) than ICV, replicating findings in several past studies (Cardenas et al., 2011; Sluimer et al., 2008). However, it is important to note that a small but significant effect of ICV on later-life cognitive ability remained in both models. The magnitudes of these associations did not differ significantly across males and females. Given the current sample size, we consider these associations to be accurate and robust. As such, the current study confirms only a very modest association of maximal brain size with cognitive ability in old age. Despite this association being weak, it does show some support for the passive cerebral reserve hypothesis, which suggests that a larger brain can endure more insults before clinical or cognitive deficits emerge (Stern, 2009), although establishing the substantive or practical importance of the association between ICV and later life cognitive ability is complex.

A large body of research has considered whether gray or white matter deterioration has the greater impact on cognitive aging (Taki et al., 2011; Ziegler et al., 2010), with mixed results. In the current study, the effects of white and gray matter volume on g were largely equal and held in both males and females, suggesting a comparable influence on later life cognitive ability. However, as has been noted previously (Ziegler et al., 2010), gray and white matter deterioration may localize in different areas of the brain, and may therefore have differentiated effects on cognition. Thus, although the associations may be comparable for both gray and white matter whole-brain volumes, the functional effect may be differentiated because of their roles in the underlying neural networks.

As previously noted, the simple correlational analyses in the current study provided a number of important estimates for the research literature on both brain size and cognition, and ICV and current brain status in aging individuals. Specifically, we found positive associations between brain volume and cognition both in youth (males, 0.28; females, 0.12), where ICV in old age was used as an indicator of brain size in youth, and later life (males, 0.27; females, 0.26). These findings are in close agreement with the previous meta-analysis by McDaniel (2005) and the literature reviews by Rushton and Ankney (2009) and Miller and Penke (2007). Our findings contribute significantly to this literature, as our single sample (N = 620, full sample; n = 537, MMSE ≥28) is as large as approximately 40%–45% of the total sample reported in these quantitative reviews. Moreover, this sample provides estimates of these effect sizes across 60 years of the life course in the same subjects.

The study has several strengths. It is rare to have access to cognitive ability scores from youth in older age. The sample is large and homogenous in terms of the age range of participants. As such, our analyses gain statistical power, and have a natural control for the confounding effect of chronological age (Hofer et al., 2006). An additional advantage of our large sample was the ability to estimate models reliably in males and females independently, rather than simply including sex as a covariate in statistical analyses. Given the large array of well-validated cognitive tests administered in older age and the large sample size, we were able to estimate all models using SEM, thus providing reliable estimates of latent cognitive ability constructs that explicitly account for measurement error, and allowing the simultaneous estimate of all substantive and covariate parameters.

A possible limitation of the current study was the assumption that ICV remains static once maximal brain size is reached in youth. Although it was not possible to test this assumption in our primary data, secondary data are available that provide justification for this assumption. Specifically, analysis of open-access brain MRI data showed that ICV remains broadly stable across the adult life course in a pooled sample from cross-sectional studies, whereas brain tissue as a percentage of ICV declines with age. However, although the presented cross-sectional data support our assumption, a full confirmation would require longitudinal imaging from youth to older age, data that, to the authors' knowledge, is currently not available.

A further concern regarding the stability of ICV across the lifespan is that the potential influence of age-related changes to the skull, such as thickening of the inner skull table (May et al., 2010), may lead to underestimates of brain changes when ICV is used as a measure of pre-morbid brain volume, especially because inner table skull thickening is known to affect women more than men. However, as of now, there are no reliable methods for estimating ICV that take account of the effect of inner skull thickening. Thus we acknowledge the possibility of bias due to such effects, but we are not able to provide any reasonable adjustments to the current findings.

In future research, we aim to study the aging process in more detail, using repeated measures of broad and specific cognitive functions and brain parameters. In the current study, our childhood estimate of cognitive ability was an overall IQ score and, as such, we focused specifically on general cognitive ability (g) in later life as the principal outcome. However, we recognize that cognitive ability is known to be constituted by several different domains, and future research with additional longitudinal data that are not currently available will be able to consider the associations between these domains and longitudinally measured brain atrophy.

The current study yields a number of important conclusions for the associations between brain status and cognitive ability in older age. First, both prior and current brain size are significant predictors of current cognitive ability, over and above the influence of prior cognitive ability. Second, the effects were highly similar for white and gray matter. These conclusions hold in both males and females.

## Disclosure statement

The authors declare that they have no actual or potential conflicts of interest with this work.

## Figures and Tables

**Table 1:** Descriptive statistics for cognitive ability and brain imaging variables in the LBC1936 (N = 620)

Characteristic	Mean		SD		Skew		Kurtosis	
	Male (n = 327)	Female (n = 293)	Male (n = 327)	Female (n = 293)	Male (n = 327)	Female (n = 293)	Male (n = 327)	Female (n = 293)
Age (y)	72.47	72.60	0.70	0.73	0.10	−0.06	−0.84	−0.85
MMSE	28.75	29.04	1.27	1.10	−0.93	−1.17	0.25	1.18
Cognitive ability Age 11 y								
Age 11 IQ	99.72	102.98	16.17	13.39	−0.90	−0.67	1.12	0.49
Cognitive ability Age 73 y								
Digit span backward	7.85	8.04	2.27	2.24	0.34	0.30	−0.03	−0.30
Block design	35.96	32.93	10.53	8.85	0.28	0.56	−0.28	0.76
Letter–number sequencing	11.07	11.07	3.06	2.74	0.36	0.44	0.32	0.51
Matrix reasoning	14.09	13.00	4.82	4.75	−0.19	−0.03	−0.89	−0.90
Digit symbol coding	54.78	58.95	12.17	11.27	0.19	0.22	−0.36	−0.16
Symbol search	24.74	25.15	6.23	5.69	−0.31	−0.16	0.55	0.93
Estimated childhood brain volume								
ICV (cm^3^)	1536.93	1355.39	113.20	101.08	0.25	0.28	−0.15	−0.13
Brain volume age 73 y								
Total brain tissue volume (cm^3^)	1175.08	1070.08	100.03	83.70	0.16	0.14	−0.07	−0.18
White matter volume (cm^3^)	522.22	468.04	84.87	68.89	0.46	0.17	0.20	0.31
Gray matter volume (cm^3^)	521.59	476.49	71.80	62.36	0.14	0.01	1.17	−0.13

Key: SD, standard deviation.

**Table 2:** Pearson's correlations between independent, dependent, and covariate variables

	1	2	3	4	5	6	7	8	9	10	11	12
1. Age	—	−0.14^∗^	−0.05	−0.03	−0.02	0.05	−0.17^∗∗^	−0.14^∗^	−0.03	−0.13^∗^	−0.23^†^	0.11
2. Age 11 IQ	−0.04	—	0.44^†^	0.40^†^	0.34^†^	0.41^†^	0.35^†^	0.35^†^	0.12^∗^	0.11	0.11	−0.01
3. Block design	−0.18^∗∗^	0.42^†^	—	0.53^†^	0.29^†^	0.31^†^	0.38^†^	0.42^†^	0.19^∗∗^	0.25^†^	0.15^∗∗^	0.07
4. Matrix reasoning	−0.12^∗^	0.41^†^	0.49^†^	—	0.33^†^	0.30^†^	0.28^†^	0.24^†^	0.09	0.14^∗^	0.14^∗^	0.01
5. Digit span backward	−0.11^∗^	0.33^†^	0.29^†^	0.35^†^	—	0.47^†^	0.20^†^	0.18^∗∗^	0.07	0.11	0.04	0.06
6. Letter–number sequencing	−0.11	0.39^†^	0.33^†^	0.38^†^	0.56^†^	—	0.27^†^	0.21^†^	0.07	0.10	0.08	0.04
7. Digit symbol	−0.16^∗^	0.46^†^	0.47^†^	0.43^†^	0.38^†^	0.51^†^	—	0.58^†^	0.11	0.22^†^	0.13^∗∗^	0.13^∗∗^
8. Symbol search	−0.13^∗^	0.41^†^	0.48^†^	0.42^†^	0.37^†^	0.41^†^	0.65^†^	—	0.06	0.17^∗∗^	0.18^∗∗^	0.04
9. ICV	−0.01	0.28^†^	0.17^∗∗^	0.11^∗^	0.14^∗∗^	0.11	0.21^†^	0.26^†^	—	0.83^†^	0.51^†^	0.42^†^
10. Brain volume	−0.13^∗^	0.26^†^	0.25^†^	0.18^∗∗^	0.23^†^	0.22^†^	0.33^†^	0.34^†^	0.82^†^	—	0.58^†^	0.52^†^
11. WM volume	−0.21^†^	0.18^∗∗^	0.18^∗∗^	0.13^∗^	0.19^∗∗^	0.24^†^	0.26^†^	0.19^∗∗^	0.51^†^	0.61^†^	—	−0.07
12. GM Volume	0.13^∗^	0.11^∗^	0.14^∗^	0.04	0.06	0.04	0.18^∗∗^	0.26^†^	0.36^†^	0.48^†^	−0.09	—

Note: Correlations for males (n = 327) are below the diagonal; correlations for females (n = 293) are above the diagonal. All correlations are uncorrected. Key: GM, gray matter; ICV, intracranial volume; WM, white matter. * *p <* 0.05; ** *p <* 0.01; ^†^*p <* 0.001.

**Table 3:** Mean estimated intracranial volume (eTIV) and percentage total brain volume of eTIV (TBV) grouped by decade, in the whole dataset and split by gender

Age group (y)	n	Male/female	Age, y, mean (SD)	eTIV (cm^3^), mean (SD)	TBV, (% eTIV) mean (SD)
18–28	135	59/76	22.07 (2.58)	1515 (150)	84.7 (1.9)
29–39	19	13/6	32.68 (3.35)	1511 (133)	83.1 (2.0)
40–50	36	12/24	46.19 (2.97)	1446 (164)	82.1 (2.3)
51–61	33	10/23	55.97 (3.11)	1462 (161)	81.0 (2.2)
62–72	63	23/40	68.27 (3.08)	1449 (138)	75.8 (4.2)
73–83	90	29/61	77.68 (3.32)	1478 (182)	73.2 (3.2)
84–96	40	14/26	88.03 (3.01)	1450 (149)	70.6 (3.4)

Data were obtained from OASIS (http://www.oasis-brains.org).
